# Long-term urodynamic evaluation of laparoscopic radical cystectomy with orthotopic ileal neobladder for bladder cancer

**DOI:** 10.3892/ol.2014.2281

**Published:** 2014-06-24

**Authors:** DONG WANG, LI-JUN LI, JING LIU, MING-XING QIU

**Affiliations:** Department of Urology, Sichuan Academy of Medical Sciences and Sichuan Provincial People’s Hospital, Chengdu, Sichuan 610072, P.R. China

**Keywords:** urodynamics, laparoscopic radical cystectomy, orthotopic ileal neobladder, bladder cancer

## Abstract

The long-term urodynamics of laparoscopic radical cystectomy with orthotopic ileal neobladder for bladder cancer remain unclear in the clinical setting. The present prospective observational study was conducted between January 2010 and December 2012 to evaluate the 6-month and 12-month follow-up data of urodynamic changes of bladder cancer patients who were initially treated by laparoscopic radical cystectomy with orthotopic ileal neobladder. A total of 53 eligible patients were included, and all patients were followed up for at least 12 months, with a median time of 18 months. During the follow-up period, no patients reported difficulty urinating, and the daily frequency of urination and the urine output were gradually improved with time. Dynamic urodynamic examinations showed that the maximum flow rate (11.4±1.1 vs. 7.3±1.4 ml/sec; P<0.001), residual urine content (22.8±10.5 vs. 40.7±12.7 ml; P<0.001), maximum bladder capacity (373.8±62.2 vs. 229.7±56.3 ml; P<0.001) and maximum bladder pressure during filling (35.8±6.7 vs. 26.4±7.0 cm H_2_O; P<0.001) at 12 months were all improved significantly compared with that at 6 months after the initial surgical treatment. However, there were no significant differences in maximum bladder pressure during voiding (75.7±24.7 vs. 73.1±24.7 cm H_2_O; P=0.618) and bladder compliance (26.9±13 vs. 27.4±13.1 cm H_2_O; P=0.848) at 12 and 6 months after initial surgical treatment. In conclusion, the urodynamics of this orthotopic ileal neobladder gradually improve, and its long-term urine storage and voiding functions are acceptable.

## Introduction

Bladder cancer is a major health problem, particularly among males. It is estimated that in the year 2008, 150,000 patients succumbed to bladder cancer and 386,300 new patients were diagnosed throughout the world (1). In the USA, bladder cancer is the fifth most common malignancy (2). According to a critical systematic review, bladder cancer is the most expensive malignancy to treat on a per-patient basis due to the requirement for lifelong routine monitoring and treatment from diagnosis to mortality (3,4).

Over the past 10 years, great progress has been made in our understanding of bladder cancer (5,6), and radical cystectomy with orthotopic ileal neobladder is frequently performed for muscle-invasive bladder cancer and high-risk superficial tumors that are unresponsive to intravesical therapy (7,8). With the development of laparoscopic surgery in urology, laparoscopic radical cystectomy with different urinary diversions has been reported as a step by step procedure (9,10). Another study has indicated that laparoscopic radical cystectomy with orthotopic ileal neobladder is feasible and provides a number of intraoperative and post-operative advantages over a traditional open approach (11), including decreasing the amount of intraoperative bleeding, reducing post-operative pain and complications, shortening the time required for recovery, and allowing low morbidity and oncological safety.

Although the surgical techniques of laparoscopic radical cystectomy with orthotopic ileal neobladder have been studied extensively, no previous study has examined the long-term urodynamic changes of this surgical procedure. Urodynamic testing or urodynamics assesses the ability of the bladder and urethra to store and release urine (12). The purpose of the present study was to determine the long-term urodynamics of laparoscopic radical cystectomy with orthotopic ileal neobladder for patients with bladder cancer. This long-term evaluation of urodynamics would also aid in promoting the recognition of this procedure as an effective standard treatment for bladder cancer.

## Patients and methods

### Patients and study design

This prospective observational study was conducted between January 2010 and December 2012. Bladder cancer patients who received laparoscopic radical cystectomy with orthotopic ileal neobladder in the Department of Urology, Sichuan Academy of Medical Sciences and Sichuan Provincial People’s Hospital (Chengdu, Sichuan, China), were included, while bladder cancer patients who showed evidence of distant metastases at diagnosis, complications with serious internal diseases or any other malignancies were all excluded.

This study was approved by the Institutional Review Board of the Sichuan Academy of Medical Sciences and Sichuan Provincial People’s Hospital and was conducted in accordance with the 1975 Declaration of Helsinki. All patients signed informed consent forms prior to participating in this study.

### Surgical procedures and urodynamic assessment

Prior to surgery, a complete physical examination and routine other examinations, including urodynamic testing and pre-operative examinations, such as cystoscopy, ultrasonography, abdominal and pelvic CT or MRI, were all performed. The laparoscopic radical cystectomy and orthotopic ileal neobladder procedures were performed by surgical specialists, and detailed information on the surgical procedures can be obtained from a previously published study (10). When discharged from hospital following surgery, all patients were prospectively followed up at a clinic in the Sichuan Academy of Medical Sciences and Sichuan Provincial People’s Hospital for >12 months, and excretory urography and urodynamics were examined every 6 months.

In this study, urodynamic evaluations were performed using a Nidoc 970 A Urodynamic System (Yongxin Medical Equipment Co., Ltd., Chengdu, Sichuan, China), and the following urodynamic parameters were assessed: Maximum flow rate, residual urine, maximum bladder capacity, maximum bladder pressures during filling and voiding, and bladder compliance.

### Statistical analysis

The storage and statistical analyses were performed using SPSS version 17.0 (SPSS, Inc., Chicago, IL, USA). Continuous data are presented as the mean ± standard deviation, while categorical data are presented as n (%). Comparisons of continuous data employed an independent two-sided paired t-test. P<0.05 was considered to indicate a statistically significant difference.

## Results

### General information

**Figure 1:**
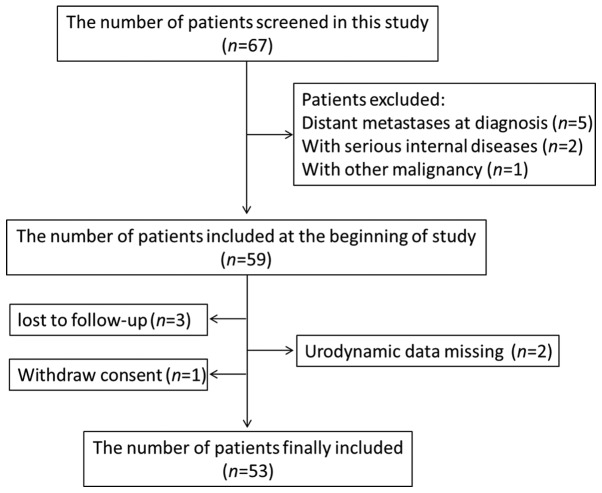
Flowchart of patient inclusion and exclusion.

As shown in Fig. 1, a total of 67 bladder cancer patients were screened in the present study, and only 53 patients were finally included; the other 14 patients were excluded from the statistical analysis for various reasons.

The detailed demographics, pre-operative surgical history and pre-operative stage parameters of the 53 included patients are all listed in Table I. The intraoperative events and short-term postoperative data of the patients are summarized in Table II, and indicated that laparoscopic radical cystectomy with extracorporeal formation of a neobladder are feasible and safe procedures for bladder cancer patients.

### Changes in urodynamics

In order to investigate the long-term efficacy of laparoscopic radical cystectomy with orthotopic ileal neobladder for bladder cancer, all patients were followed up for at least 12 months and the urodynamic improvement of the patients was evaluated. During the follow-up period, no patients reported difficulty in urinating (Table II), and the daily frequency of urination and the urine output gradually improved with time.

**Figure 2:**
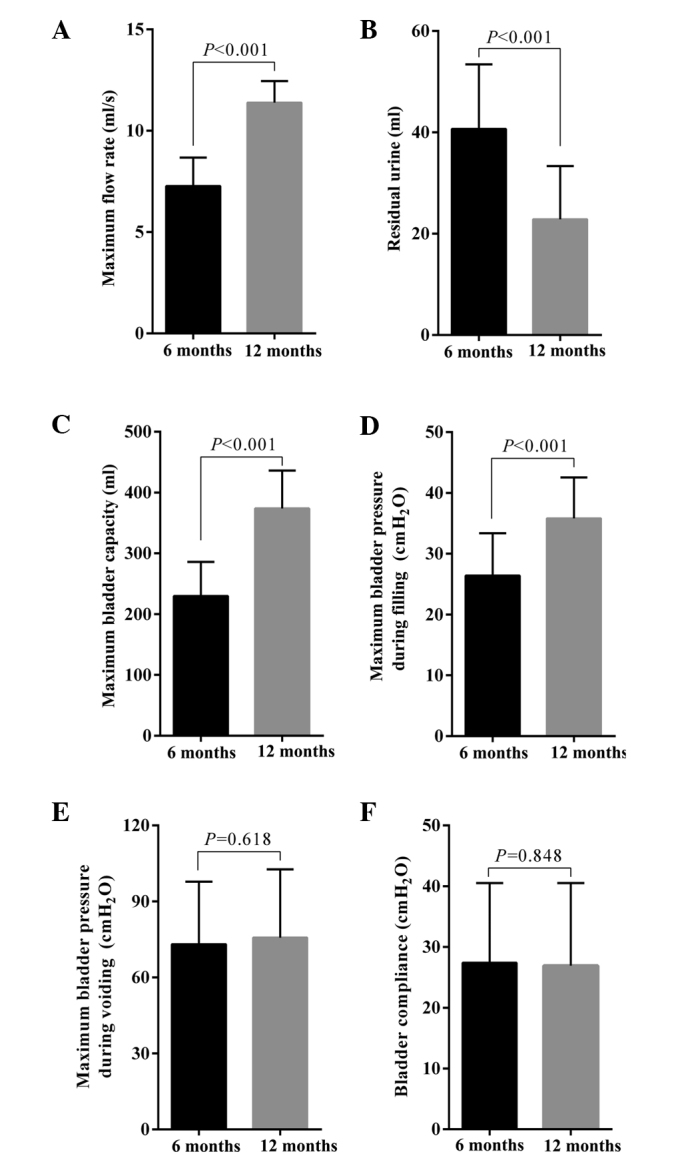
Urodynamic test results at 6 and 12 months post-surgery. (A) Maximum flow rate; (B) residual urine; (C) maximum bladder capacity; (D) maximum bladder pressure during filling; (E) maximum bladder pressures during voiding; and (F) bladder compliance.

As shown in Fig. 2, there was a time-related continuous improvement in urodynamics. Compared with 6 months after the initial surgical treatment, the maximum flow rate (11.4±1.1 vs. 7.3±1.4 ml/sec; P<0.001), residual urine (22.8±10.5 vs. 40.7±12.7 ml; P<0.001), maximum bladder capacity (373.8±62.2 vs. 229.7±56.3 ml; P<0.001) and maximum bladder pressure during filling (35.8±6.7 vs. 26.4±7.0 cm H_2_O; P<0.001) at 12 months had all improved significantly. However, there was no significant difference in either the maximum bladder pressures during voiding (75.7±24.7 vs. 73.1±24.7 cm H_2_O; P=0.618) or bladder compliance (26.9±13.6 vs. 27.4±13.1 cm H_2_O; P=0.848) at 12 and 6 months after initial surgical treatment. These long-term dynamic improvements in urodynamics showed that the constructed neobladder had good storage and voiding functions.

## Discussion

Bladder cancer is one of the most common malignancies, and has become the most expensive to treat on a per-patient basis (13). Due to the extreme severity and metastasis-prone nature, bladder cancer patients have a poor prognosis and high mortality rate. Although the treatment of bladder cancer is complex, surgery remains generally acknowledged as the most effective treatment (14). Currently, radical cystectomy is widely used for muscle-invasive bladder cancer treatment, particularly for bladder cancer with T-staging >2. With the continuous improvement of surgical techniques and the wide application of advanced auxiliary equipment, there have been a number of studies documenting novel surgical approaches for bladder cancer (15–17), including laparoscopic radical cystectomy with orthotopic ileal neobladder, and due to the successful reconstruction of the neobladder, the post-operative quality of life of these patients has been greatly improved in recent years.

Although urinary diversion following radical cystectomy can be carried out in several ways, orthotopic neobladder has been recognized as a relatively effective surgery (19). Besides avoiding the occurrences of urine and stool mixing and urine extravasation, the physiological function of the orthotopic bladder is also extremely close to the original bladder compared with other traditional urinary diversion surgeries (20). Considering the functional association between the bladder and bowel, the potent clinical application values of orthotopic ileal neobladder following radical cystectomy have been widely assessed (21–23). Currently, a program of strictly timed voiding has been carried out in all post-operative patients, which aids patients in establishing the storage and voiding functions of new bladders as soon as possible. However, significant differences remain in the biological characteristics and neural induction between the ileum and bladder. Long-term dynamic monitoring of neobladder function following surgery is important, and thus provides a basis for timely clinical intervention if abnormalities of the neobladder observed.

A normal lower urinary tract should be able to achieve efficient and low-pressure bladder filling, low-pressure urine storage with perfect continence and periodic complete voluntary urine expulsion, again at low pressure (24). In the present study, the orthotopic ileal bladder that was constructed exhibited a high capacity, improved compliance and low pressure at 6 months post-surgery. The residual urine content was also gradually reduced with the extension of the post-operative recovery time, which significantly contributed to the prevention of urinary tract infections. Moreover, the aforementioned functional parameters of the orthotopic ileal bladder were further improved at 12 months post-surgery. Thus, these findings further indicate the technical feasibility and improved long-term prognosis of the orthotopic ileal bladder, which is consistent with a previous study (25).

Studies have reported that the occurrence of night continence is ~20%, which is higher than that of day continence. Although the mechanism of night continence is not fully clarified, pelvic muscle relaxation, contraction of external sphincter and reservoir allantoic, and the lack of sensing urine expansion may be possible causes (26). In the present cohort, the ideal improvement in urodynamics during long-term follow-up may also be explained by the adequate level of health education and early bladder voiding function training, including timed voiding during the day and night, a balanced intake of water throughout the day and increased abdominal pressure using the hands (or a squatting posture) to maximize emptying of the bladder when urinating.

The limitation of the present study is the relatively small sample size, and consequently, large sample, multi-center studies are required. However, the dynamic monitoring data for the urodynamics reported in this study provide novel valuable evidence for the comprehensive evaluation of laparoscopic radical cystectomy with orthotopic ileal neobladder for bladder cancer in real life clinical practice.

## Figures and Tables

**Table I:** Demographics and pre-operative clinical parameters.

Parameters	Value
Demographics	
Age, years	58.7±7.3
Male/female, n (%)	51 (96.2)/2 (3.8)
Body mass index, kg/m^2^	20.9±2.7
History of open or abdominal/pelvic surgery, n (%)	
Partial cystectomy	6 (11.3)
Cholecystectomy	3 (5.7)
Repair of gastric perforation	2 (3.8)
Partial gastrectomy	2 (3.8)
Appendectomy	1 (1.9)
Pre-operative clinical T-staging, n (%)	
T1	6 (11.3)
T2a	28 (52.8)
T2b	10 (18.9)
T3b	8 (15.1)
T4a	1 (1.9)
Pre-operative clinical N-staging, n (%)	
N0	49 (92.5)
N1	4 (7.5)
Pre-operative histological characteristics, n (%)	
Grade I/II/III	7 (13.2)
Grade II	42 (79.2)
Grade III	4 (7.5)

**Table II:** Intraoperative events and post-operative data.

Parameters	Value
Duration of surgical procedures, min	308.5±62.7
Estimated blood loss, ml	210.5±106.8
Blood transfusion, n (%)	8 (15.1)
Duration of intra-abdominal drainage, days	3.2±1.4
Duration of foley catheter, days	12.4±2.9
Duration of bilateral ureteral stent, days	11.8±2.2
Bowel activity recovery time post-surgery, days	3.1±1.8
Time until food intake post-surgery, days	4.2±0.7
Hospitalization time post-surgery, days	13.5±2.6
Follow-up time post-discharge, months	17.6±5.3
Difficulty urinating during follow-up, n (%)	0 (0.0)
